# The β-blocker Nebivolol Is a GRK/β-arrestin Biased Agonist

**DOI:** 10.1371/journal.pone.0071980

**Published:** 2013-08-20

**Authors:** Catherine E. Erickson, Rukhsana Gul, Christopher P. Blessing, Jenny Nguyen, Tammy Liu, Lakshmi Pulakat, Murat Bastepe, Edwin K. Jackson, Bradley T. Andresen

**Affiliations:** 1 Department of Internal Medicine, University of Missouri, Columbia, Missouri, United States of America; 2 Department of Nutrition and Exercise Physiology, University of Missouri, Columbia, Missouri, United States of America; 3 Department of Medical Pharmacology and Physiology, University of Missouri, Columbia, Missouri, United States of America; 4 Harry S Truman Memorial Veterans’ Hospital, Columbia, Missouri, United States of America; 5 Obesity Research Center, College of Medicine, King Saud University, Riyadh, Saudi Arabia; 6 Endocrine Unit, Department of Medicine, Massachusetts General Hospital and Harvard Medical School, Boston, Massachusetts, United States of America; 7 Department of Pharmacology and Chemical Biology, University of Pittsburgh, Pittsburgh Pennsylvania, United States of America; 8 Department of Pharmaceutical Sciences, Western University of Health Sciences, Pomona, California, United States of America; Cornell University, United States of America

## Abstract

Nebivolol, a third generation β-adrenoceptor (β-AR) antagonist (β-blocker), causes vasodilation by inducing nitric oxide (NO) production. The mechanism via which nebivolol induces NO production remains unknown, resulting in the genesis of much of the controversy regarding the pharmacological action of nebivolol. Carvedilol is another β-blocker that induces NO production. A prominent pharmacological mechanism of carvedilol is biased agonism that is independent of Gα_s_ and involves G protein-coupled receptor kinase (GRK)/β-arrestin signaling with downstream activation of the epidermal growth factor receptor (EGFR) and extracellular signal-regulated kinase (ERK). Due to the pharmacological similarities between nebivolol and carvedilol, we hypothesized that nebivolol is also a GRK/β-arrestin biased agonist. We tested this hypothesis utilizing mouse embryonic fibroblasts (MEFs) that solely express β_2_-ARs, and HL-1 cardiac myocytes that express β_1_- and β_2_-ARs and no detectable β_3_-ARs. We confirmed previous reports that nebivolol does not significantly alter cAMP levels and thus is not a classical agonist. Moreover, in both cell types, nebivolol induced rapid internalization of β-ARs indicating that nebivolol is also not a classical β-blocker. Furthermore, nebivolol treatment resulted in a time-dependent phosphorylation of ERK that was indistinguishable from carvedilol and similar in duration, but not amplitude, to isoproterenol. Nebivolol-mediated phosphorylation of ERK was sensitive to propranolol (non-selective β-AR-blocker), AG1478 (EGFR inhibitor), indicating that the signaling emanates from β-ARs and involves the EGFR. Furthermore, in MEFs, nebivolol-mediated phosphorylation of ERK was sensitive to pharmacological inhibition of GRK2 as well as siRNA knockdown of β-arrestin 1/2. Additionally, nebivolol induced redistribution of β-arrestin 2 from a diffuse staining pattern into more intense punctate spots. We conclude that nebivolol is a β_2_-AR, and likely β_1_-AR, GRK/β-arrestin biased agonist, which suggests that some of the unique clinically beneficial effects of nebivolol may be due to biased agonism at β_1_- and/or β_2_-ARs.

## Introduction

Nebivolol is classified as a third generation β-adrenoceptor (β-AR) antagonist (β-blocker) that has a higher affinity for β_1_-adrenoceptors (β_1_-ARs) compared to β_2_-ARs and β_3_-ARs [1], [2]. Importantly, nebivolol activates endothelial nitric oxide (NO) synthase (eNOS) leading to vasorelaxation *in vivo* and *ex vivo* [3]–[5]. Although the mechanism is unknown, a leading theory is that nebivolol induces vasodilation via β_3_-ARs [5]–[8]. However, recent evidence argues against a mechanism involving human β_3_-ARs [9], and nebivolol’s reported affinity for β_3_-ARs (K_i_ ∼ 1.5 µM) is much weaker than for β_2_-ARs (K_i_ ∼ 30 nM) and β_1_-ARs (K_i_ ∼ 0.8 nM) [9], [10]. Therefore, it seems unlikely that pharmacologically relevant concentrations of nebivolol would act via β_3_-ARs, especially *in vivo* where 98% of nebivolol is bound to plasma proteins [11]. Because of the ratios of agonist to antagonist utilized the most recent reports regarding nebivolol acting through β_3_-ARs [5] leaves open the possibility that nebivolol could act, at least in part, via β_1_-ARs or β_2_-ARs. The β_3_-AR is not the only theorized target receptor; an alternative theory is that metabolites of nebivolol induce vasodilation through β_2_-ARs [12].

Nebivolol abolishes β_1_- and β_2_-AR-mediated cAMP generation in response to classical agonists and does not generate cAMP through either receptor on its own [9], [13], [14]. Because of these antagonistic properties at β_1_- and β_2_-ARs, the theories described previously have grown in acceptance due to viewing nebivolol solely as a classic antagonist. However, the recently developed concept of biased agonism indicates that classification based on cAMP production alone is not sufficient to dismiss a ligand from having agonistic properties at β_1_- and β_2_-ARs.

Biased agonism is a relatively new term that defines a subset of functional selectivity [15]–[17]. G protein-coupled receptors (GPCRs) signal through at least two mechanisms: the traditional Gα and Gβγ pathways, as well as the more recently appreciated G protein-coupled receptor kinase (GRK)/β-arrestin pathway. Traditional agonists signal through both of these mechanisms simultaneously, and pure antagonists block both pathways simultaneously; however, biased agonists favor one mechanism over the other. This bias allows for a ligand to signal differently than a traditional agonist and, importantly, means that what once were called “antagonists” (because they inhibit the Gα and Gβγ pathways) may, in fact, activate the GRK/β-arrestin pathway, which would result in unique properties compared to pure antagonists.

Thus far, there is only one known clinically utilized GRK/β-arrestin biased agonist: carvedilol [18], [19]. Carvedilol, which is now available as a generic drug, was originally marketed as a β-blocker. In 2007, carvedilol was recognized as a biased agonist that induces β-AR-mediated activation of the GRK/β-arrestin pathway but not the Gα_s_-coupled/cAMP pathway [19]–[21]. Furthermore, carvedilol along with the other third generation β-blockers, such as nebivolol, are unique among β-blockers because they result in vasodilatation. Like nebivolol, carvedilol-mediated vasodilation is attributed to nitric oxide (NO) production [22]. Moreover, carvedilol-mediated NO production and vasodilation may be due, at least partially, to biased agonism at β-ARs [23].

Given the similarities between carvedilol and nebivolol, it is possible that they signal through similar mechanisms. Although studies have examined most β-blockers for biased-agonist activity [19], [20], to date nebivolol has not been examined. Nebivolol is an intriguing candidate for GRK/β-arrestin biased agonism because of its similarities with carvedilol and its debated signaling mechanisms. Since vasodilation can be mediated through an endothelial cell epidermal growth factor receptor (EGFR)-mediated mechanism [24]–[26], β-AR-mediated transactivation of the EGFR through a GRK/β-arrestin biased signaling mechanism could explain nebivolol-mediated NO production without invoking the need for additional ligand-receptor interactions such as via β_3_-ARs. Therefore, we tested the hypothesis that nebivolol is a GRK/β-arrestin biased agonist.

Assessment of GRK/β-arrestin biased agonism can be accomplished through various approaches including phosphorylation of the receptor, receptor internalization, redistribution of β-arrestins, and β-arrestin-mediated signaling. To avoid inducing any over-expression artifacts, we utilized endogenous β-ARs which, due to poor β-AR specific antibodies [27], limited these studies to: β-AR internalization and signaling; β-arrestin-mediated signaling and cellular redistribution; as well as probing for the role of the EGFR and GRKs. Data generated by these studies collectively indicate that nebivolol is indeed a GRK/β-arrestin biased agonist.

## Methods

### Chemicals

Nebivolol was obtained from Forest Laboratories as well as purchased from Tocris (Bristol, England, UK) and Sigma (St. Louis, MO). Norepinephrine, isoproterenol, propranolol, alprenolol, 3-isobutyl-1-methylxanthine (IBMX), and forskolin were obtained from Sigma; *N*-(3-chlorophenyl)-6,7-dimethoxy-4-quinazolinanine hydrochloride (AG1478) and methyl 5-[2-(5-nitro-2-furyl)vinyl]-2-furoate (GRK2 inhibitor) was obtained from EMD Millipore/Calbiochem (San Diego, CA); and [5,7-^3^H] (-)-CGP-12177 (chemical name: 4-[3-[(1,1-dimethylethyl)amino]2-hydroxypropoxy]-1,3-dihydro-2*H*-benzimidazol-2-one hydrochloride), henceforth referred to as ^3^H-CGP, was obtained from Perkin Elmer (Waltham, MA). Hoechst 33342 was obtained from Life Technologies (Carlsbad, CA). All other materials are described below.

### Cell Culture

Mouse embryonic fibroblasts lacking all the *Gnas* exon 2 derived products including Gα_s_ and XLα_s_ (*Gnas* ^E2−/E2−^) have been described previously [28]. These cells were stably transfected with a plasmid containing human Gα_s_ short driven by an EF-1 promoter and conferring blasticidin resistance. Functional expression of Gα_s_ and β-ARs was confirmed by measuring cAMP production in the presence and absence of 10 µM isoproterenol (data not shown). For simplicity, these cells are referred to as MEFs in this manuscript. The *Gnas* ^E2−/E2−^ cells and MEFs were grown in DMEM-F12 supplemented with 10% FBS, 50 units of penicillin-streptomycin (Life Technologies), and 5 µg/mL blasticidin from InvivoGen; San Diego, CA (for the MEFs only). These cells were maintained at 37°C in 5% CO_2_ plus 95% air humidified incubator.

Mouse atrial myocytes (HL-1) were a gift from Dr. William Claycomb (Louisiana State University, New Orleans, LA, USA) [29]. The myocytes were cultured in complete Claycomb supplemented with 10% FBS (JRH Biosciences, Lenexa, KS), penicillin-streptomycin (100 U/ml), norepinephrine (100 U/ml) and L-glutamine (2 mM) (Life Technologies). The culture flasks were pre-coated with 12.5 µg/ml fibronectin in 0.02% gelatin solution (Sigma). The myocytes were also maintained at 37°C in 5% CO_2_ plus 95% air humidified incubator.

### Identification of β-ARs and GRKs

Taq-Man qPCR was utilized to identify the β-AR and GRK isoforms expressed in MEFs; HL-1 cells and mouse aorta total RNA (positive control) were also used to identify the β-ARs. The primers were designed utilizing the online primer designer from IDT (Coralville, IA) to amplify mouse β_1_-, β_2_-, and β_3_-ARs, and GRK 2, 3, 4, 5, and 6; additionally a universal 18 s primer was used as an internal control (Table 1). Qiagen (Valencia, CA) RNeasy Plus Mini kit with shredder columns were used as directed to isolate total RNA from confluent plates of cells, and the total RNA was converted to cDNA using Qiagen’s Omniscript kit with RNase inhibitor from Promega (Madison, WI). The cDNA, primers, and TaqMan Universal PCR Master Mix (Life Technologies) were placed into 0.2 mL AB MicroAmp tubes from Life Technologies and the samples run on an ABI 7000 with standard cycling (10 minute 95°C followed by 40 cycles of 15 sec 95°C denaturing step and a 1 minute 60°C anneal/extend step). Each sample was run in triplicate for each of the aforementioned transcripts. An auto baseline was used to determine the Ct for all samples, and ΔCt was determined by subtracting the 18 s from the paired transcripts. The relative quantification (RQ) method utilizing 2 ^(−ΔΔCt)^ was used to compare the transcripts expression levels. For β-ARs in MEFs and HL-1 cells, the pooled ΔCt of β_1_- and β_2_-ARs of mouse aorta was used as the control in the ΔΔCt method, and for the GRKs in MEFs the β_2_-AR was used as the control. Nonparametric statistics were used to analyze the data.

**Table 1:** qPCR primers.

Target	Primer	Sequence (5′ to 3′)
18 s	Forward	CGGACAGGATTGACAGATTG
	Reverse	CAAATCGCTCCACCAACTAA
	Probe	6-Fam-CACCACCAC(Zen)CCACGGAATCG-IBFQ
ADRB1	Forward	ACTTCGGTAGATGTGCTGTG
	Reverse	AAACTCTGGTAGCGAAAGGG
	Probe	6-Fam-AGGGTCTCA(Zen)ATGCTGGCCGT-IBFQ
ADRB2	Forward	CATGGAAGGCTTTGTGAACTG
	Reverse	GTCTGGTTAGTGTCCTGTCAAG
	Probe	6-Fam-AGTCAACGC(Zen)TAAGGCTAGGCACAG-IBFQ
ADRB3	Forward	TGATGGCTATGAAGGTGCG
	Reverse	AAAATCCCCAGAAGTCCTGC
	Probe	6-Fam-CGTGAAGGG(Zen)CCGTGAAGATCCA-IBFQ
GRK1	Forward	ATTGTGTCTCTGGCCTATGC
	Reverse	CTGTGTAATAGATGGCCCGTG
	Probe	6-Fam-AATGTGGAT(Zen)GAGGATAACCCCGGC-IBFQ
GRK2	Forward	TCTTCCAGCCATACATTGAGG
	Reverse	TCATGGTCAGGTGGATGTTG
	Probe	6-FAM-ACAAGTTCA(Zen)CACGGTTCTGCCAGT-IBFQ
GRK3	Forward	GATAGACCGAATGACCCTGAC
	Reverse	ATGTGCTCCTTCAACTCTCG
	Probe	6-FAM-CAGCTTCCA(Zen)GATGCCTTCTCCCC-IBFQ
GRK4	Forward	ACCTCCAGATATTGTGAAAGAGTG
	Reverse	GCTGGCATTTGTGTTCTCTTAG
	Probe	6-FAM-CGAAGAATG(Zen)TGCTGGGTTTTCTGTCTCC -IBFQ
GRK5	Forward	GAGAAGGTTAAGCGGGAAGAG
	Reverse	TTCGAGTCTTTGGTGAGCAG
	Probe	6-FAM-AGAGGCCAA(Zen)GTCCATCTGCAACA-IBFQ
GRK6	Forward	GAGCTTCGACTCAGCCTTG
	Reverse	CATATTCAGACACCCCATCCAG
	Probe	6-FAM-CGCCTGTTA(Zen)TTTCGTGAGTTCTGTGC-IBFQ

6-FAM is the fluorescent indicator, Zen is an internally inserted dark quencher, and Iowa Black FQ (IBFQ) is a second dark quencher.

### Measurement of cAMP, Purines, and Pyrimidines

MEFs were grown in 12-well plates to near confluence and serum starved overnight in 750 µL serum free DMEM/F12. All cells were treated with 500 µM IBMX for 5 minutes, then 1 µM isoproterenol or 10 µM forskolin was added to the cells. After a 30-minute incubation at 37°C, cells were washed with ice-cold phosphate buffered saline (PBS) and 500 µL ice-cold 1-propanol was added to the cells. The cells were incubated for two hours at 4°C, and then the propanol was collected in 1.9 mL tubes and dried in a rotary evaporator. While the samples were drying, the fixed cells were stained with 5 µM of the cell permeant Hoechst 33342 dye in a 3% bovine serum albumin from Jackson ImmunoResearch (West Grove, PA) in PBS for 1 hour. The cells were then washed 3 times in PBS and each plate was imaged 3 times in a BioTek Synergy HT to account for cell number as previously described [30]. The dried samples were reconstituted in 200 µL ultrapure water and analyzed on a Thermo Electron TSQ Quantum-Ultra system with a HESI source utilizing an Agilent Zorbax eclipse XBD-C-18 column as described previously [31].

### β-AR Internalization

A modified protocol from Seibold et al. was utilized [32]. Twelve -well plates were coated with poly-D-lysine (Sigma) and cells were immediately seeded on the plates. Once confluent the cells were starved overnight in 750 µL DMEM/F12. For treatment a 4x solution of agonist (nebivolol or isoproterenol) in DMEM/F12 was prepared and 250 µL was added to each well. Two sets of zero time points were used in each experiment. When the incubation time expired, plates were placed on ice and washed 3 times with 1 mL ice cold DMEM/F12. After washing the cells, 1 mL of DMEM/F12 containing 5 nM of the cell impermeant ^3^H-CGP [33] and 5 µM Hoechst 33342 was added to all but the second zero time point. The second zero time point acted as a control for nonspecific binding; its media contained 5 nM ^3^H-CGP, 5 µM Hoechst 33342, plus 1 mM alprenolol. The dishes were then incubated for 1 hour at 4°C after which the solution was removed and the samples washed twice with ice cold DMEM/F12 and once with ice cold PBS. The PBS was removed and the plate imaged to determine the intensity of the Hoechst stain in a BioTek FLx800 fluorometer 3 times. The cells were removed from the plate by adding 200 µL trypsin/EDTA (Life Technologies) for at least 30 minutes at 37°C. All of the solution was removed from the wells and placed into scintillation vials; 5 mL scintillation fluid was added to the vials and the ^3^H-CGP counted on a Beckman LS 6000IC scintillation counter. Cell surface receptors were determined by the ratio of ^3^H-CGP/Hoechst; Hoechst 33342 staining intensity is directly related to cell number [30]. The nonspecific binding was then subtracted from each individual point and data were expressed as percent of total surface receptors at time zero. Data were fit to and analyzed with a one-phase decay model in GraphPad Prism 5 for Windows.

### Western Blots

MEFs and HL-1 cells were grown in 60 mm and 100 mm tissue culture plates, respectively, serum starved overnight (at least 12 hours) then either not pretreated (for nebivolol signaling experiments) or pretreated for 30 minutes with DMSO (vehicle), 1 µM AG1478, or 30 µM propranolol. Following treatment, cells were stimulated with an agonist (nebivolol, carvedilol, or isoproterenol) for up to 30 minutes, then lysed in lysis buffer (1% Triton X-100, 100 mM NaCl, 20 mM Tris, pH 7.5, 2 mM EDTA, 10 mM MgCl_2_, 10 mM NaF, 40 mM β-glycerol phosphate, supplemented with 1 mM PMSF and 2 mM Na_3_VO_4_). The samples were clarified by centrifuging the lysate at 14,000 rpm for 5 minutes at 4°C and removing the supernatant, then Laemmli sample buffer was added and the samples were boiled and stored at -20°C before analysis. The samples were separated on a 9% SDS-PAGE gel and transferred to nitrocellulose obtained from BioRad (Hercules, CA). ERK activation was measured via running duplicate gels and blotting with phospho-specific ERK1/2 (E10) antibodies on one blot and for total ERK1/2 on the second blot. When examining β-arrestins an additional gel (triplicate) was run and probed with β-arrestin 1/2 (D24H9) antibody. To examine GRK expression level the blots were probed with GRK specific antibodies and normalized to actin. The ERK and β-arrestin antibodies were from Cell Signaling Technology (Beverly, MA) and the GRK and actin antibodies were from Santa Cruz Biotechnology (Santa Cruz, CA); the HRP-conjugated secondary antibodies were obtained from Jackson ImmunoResearch Laboratories, and ECL was obtained from Pierce (Rockford, IL). The images were captured and analyzed using a BioRad ChemiDoc XRS system running quantity one software or a Kodak 1500 system running Carestream software.

### siRNA Knockdown of β-arrestin 1 and 2

Pooled mouse specific siRNA towards β-arrestin 1 (βarr1) (sc-29742) and β-arrestin 2 (βarr2) (sc-29743), as well as non-targeting siRNA-A and fluorescein conjugated control siRNA-A were obtained from Santa Cruz Biotechnology. The siRNA was diluted per the manufacturer’s specifications and transfected into the MEFs utilizing Life Technologies siPORT Amine following the manufacturer’s instructions except that the cells were allowed to remain attached to the tissue culture dish. The final concentration of siRNA transfected into the cells was 50 nM. The transfection media was left in the cells for two days, then the cells were serum starved overnight and the procedures under the western blot section were followed. Transfection efficiency was examined utilizing the fluorescein conjugated control siRNA-A with a Zeiss Axiovert 40 fluorescent microscope.

### β-arrestin Redistribution

MEFs were prepared for imaging as described previously [34]. Nebivolol (10 µM) was used to stimulate the cells for up to 30 minutes. The primary antibodies βarr1 (sc-9182 K-16) and βarr2 (sc-13140 H-9) from Santa Cruz Biotechnology were used at 1∶200 dilution, the fluorophore labeled secondary antibodies Alexa Fluor 555 Donkey Anti-Goat IgG for βarr1 as well as Alexa Fluor 647 Donkey Anti-Mouse IgG for βarr2 were used at a 1∶250 dilution when conducting dual staining. βarr1 did not generate a strong signal over background; therefore, the replicates utilized single staining with the βarr2 antibody and utilized 1∶250 Alexa Fluor 488 Donkey Anti-Mouse IgG as a secondary antibody. 5 µM Hoechst 33342 was used to identify the nuclei. The images were acquired with a Leica SPE confocal microscope utilizing a HCX PL APO CS 100x 1.4 NA oil objective and 405, 561, and 635 nm laser lines. All images were acquired at a resolution of 1024×1024 with identical settings for each image, and each image is an average of 3 repetitive scans. For analysis of βarr2 redistribution and accumulation in vesicles, ImageJ was utilized to identify the punctate staining that represents aggregated βarr2. Aggregation creates a greater intensity of the pixels than diffuse staining; therefore, the aggregates were identified and counted utilizing a filter to only display pixels that are greater than or equal to 64 (25% of full intensity), 96 (38% of full intensity), 128 (50% of full intensity), and 192 (75% of full intensity) intensity units. The identified punctate staining was then manually counted for each cell, and the following formula was used to integrate the number of vesicles with the threshold where the vesicles were visible: (# only seen at 64 intensity units * 64/255)+(# only seen at 96 intensity units * 96/255)+(# only seen at 128 intensity units * 128/255)+(# only seen at 192 intensity units * 192/255). The original data was obtained from 4 to 5 individual images of a cell; to increase the sample size for analysis by nonparametric statistics, resampling was used to create 8 separate sets of data. The means from each set were used in the analysis. Following analysis the image intensity was enhanced in Photpshop utilizing a sigmoidal green curve to improve visibility of the images; each image was enhanced equally.

### Statistics and Data Analysis

All graphs were generated with GraphPad Prism 6, and the Pearson’s correlation and two-way ANOVAs were also performed in GraphPad. One-way ANOVAs with Tukey-Kramer post hoc tests for normal data sets and Kruskal-Wallis posthoc test for non-normal data sets were conducted with NCSS 2007; the post-hoc tests are indicated in each figure legend. The data in the figures are expressed as mean ± SEM; textual data utilizes confidence intervals and standard deviation as detailed in the text.

## Results and Discussion

**Figure 1:**
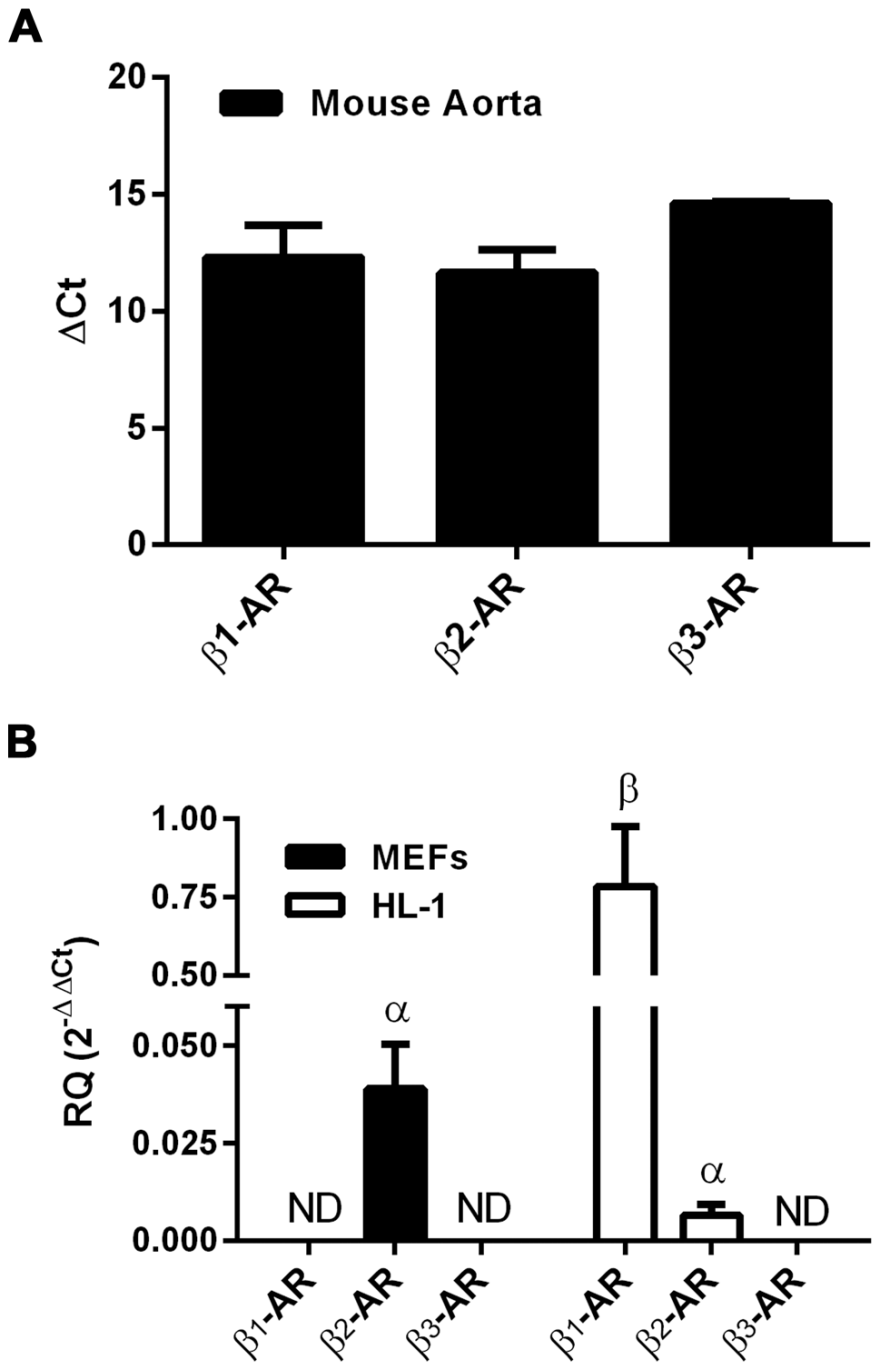
qPCR detection of β_1_-, β_2_-, and β_3_-ARs in mouse cells and tissue. Total RNA was isolated from mouse aorta (A), MEFs, and HL-1 cells (B) and subjected to 40 rounds of Taq-Man qPCR. Bars with different Greek letters are statistically different (p<0.05) from each other according to a one-way ANOVA followed by Kruskal-Wallis posthoc test. ND indicates that the transcript was not detected; data are expressed as mean ± SEM, n = 3 for cells and n = 2 for aorta.

As there is still debate regarding the role of the β_3_-AR in nebivolol-mediated signaling, the expression of β-ARs in MEFs and HL-1 cardiac myocyte cells were examined via qPCR. Since both of these cells originate from mice, and mouse aorta expresses all three subtypes of β-ARs [35], [36], mouse aorta was used as a positive control. The primer pairs were able to amplify all β-ARs subtypes in the mouse aorta (Fig. 1A) and no statistical difference was observed between the receptors although the β_3_-AR tended to have lower expression. β_1_-ARs were found only in the HL-1 cells, and β_2_-ARs were found in both MEFs and HL-1 cells (Fig. 1B). β_3_-AR message was below detection levels in both MEFs and HL-1 cells even after 40 cycles of PCR under the conditions used in this study. Expectedly, the cardiac myocyte HL-1 cells contained significantly more β_1_-AR message than β_2_-AR message; in HL-1 cells the β_1_-AR crossed the threshold ∼8 cycles before the β_2_-AR, which corresponds to approximately 256-fold greater expression of β_1_-AR mRNA compared to β_2_-AR mRNA. Importantly, these data indicate that none of the following results are likely to be attributed to the β_3_-AR, and the MEFs allow for examining predominately, if not only, β_2_-ARs.

**Figure 2:**
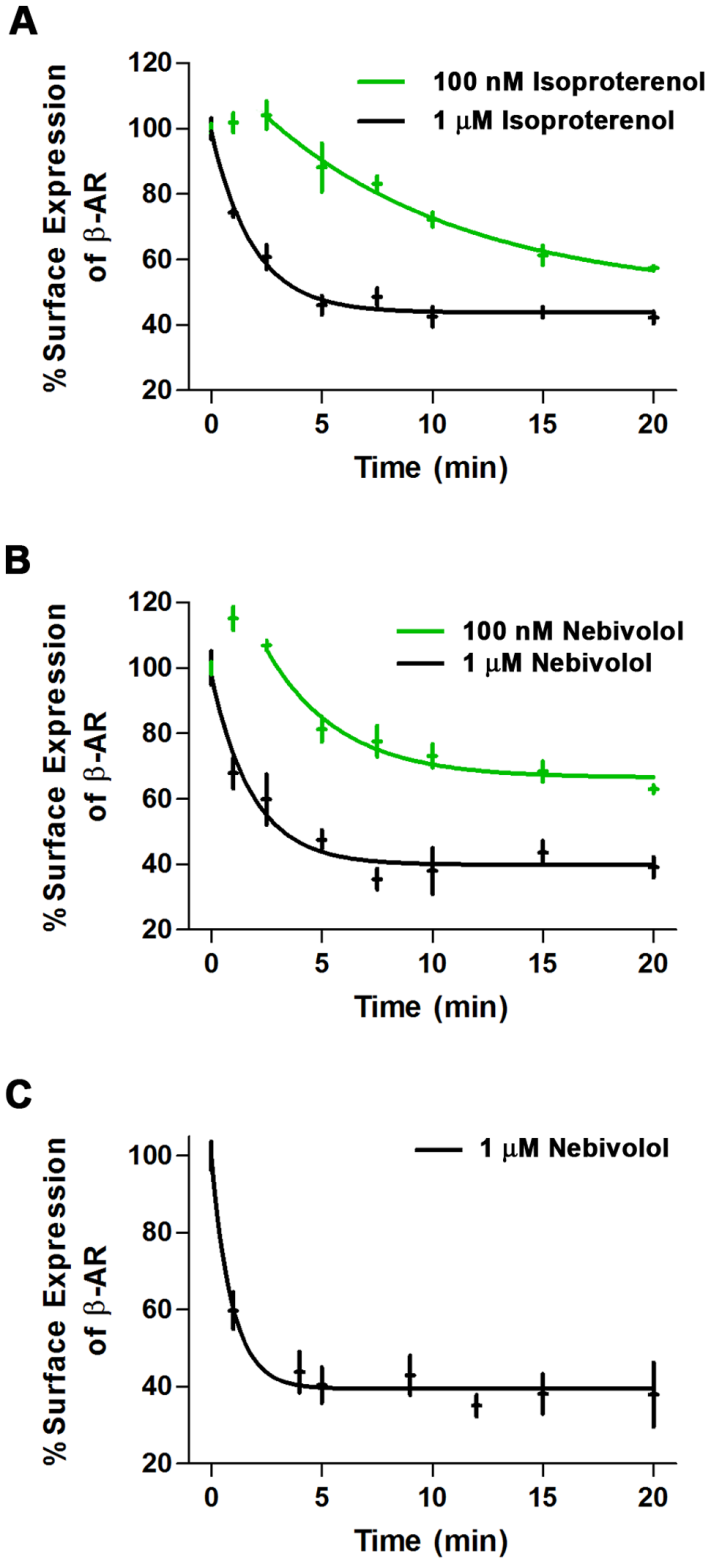
Ligand-mediated β-AR internalization. MEFs (A and B) and HL-1 cells (C) were stimulated for the indicated amount of time with isoproterenol (A) or nebivolol (B and C), and surface receptor levels were determined as described in the methods. Two doses of isoproterenol (A) and nebivolol (B) were used in the MEFs: 100 nM (green) and 1 µM (black). Data are expressed as mean ± SEM (in both the x and y axis); n = 3 to 4.

As the GRK/β-arrestin pathway is classically involved in receptor internalization and ligands that activate the GRK/β-arrestin pathway induce receptor internalization, we examined whether nebivolol stimulation resulted in internalization of β-ARs as a first test of biased agonism. Before examining the effect of nebivolol on β-AR internalization, we validated the internalization assay and determined the rates of isoproterenol-mediated β-AR internalization utilizing ^3^H-CGP. CGP is a cell impermeant β_1_- and β_2_-AR antagonist and a β_3_-AR agonist [33], [37]; however, it has much lower affinity for β_3_-ARs compared to the β_1_- and β_2_-ARs [38] and there are no detectable β_3_-ARs in the cells studied. Therefore, the observed effects cannot be attributed to β_3_-ARs. MEFs treated with 100 nM and 1 µM isoproterenol displayed a time and concentration dependent internalization of the β_2_-AR (Fig. 2A); since MEFs only express β_2_-ARs, we attribute the observed effects in MEFs to β_2_-ARs. There was at least a 2.5 minute time delay in the 100 nM isoproterenol-mediated β_2_-AR internalization; however, once internalization was initiated it progressed with a t½ of 6.260 min (95% confidence interval (CI): 3.516 to 28.510 min). The delay in internalization and the rate were concentration dependent; 1 µM isoproterenol induced internalization without any delay and resulted in a statistically shorter t½ of 1.315 min (95% CI: 1.046 to 1.769 min; p<0.05 vs. 100 nM) representing a greater rate of internalization. Similarly, 10 µM of isoproterenol (not shown) had no delay and an even more rapid rate of internalization with a t½ of 0.6928 min (95% CI: 0.5347 to 0.9836 min, p<0.05, vs. 1 µM). In all cases the plateau of the model, which represents the equilibrium between receptor internalization and recycling to the surface, was approximately 40% of the total surface receptor. Following nebivolol treatment, similar results were observed. 100 nM nebivolol had no effect for at least 2.5 minutes, but then the β_2_-AR internalized with a t½ of 2.301 min (95% CI: 1.481 to 5.162 min). 1 µM nebivolol induced internalization of the β_2_-AR with a t½ of 1.302 min (95% CI: 0.8900 to 2.422 min) (Fig. 2B). Although the rates were not different in a statistically significant manner, the plateaus were statistically different; 66.46% (95% CI: 60.48 to 72.44%) of receptors remained on the surface versus 39.81% (95% CI: 34.59 to 45.04%) in the 100 nM and 1 µM nebivolol treated samples, respectively (p<0.05). We also examined the effect of nebivolol on the β_1_- and β_2_-ARs in HL-1 cells (Fig. 2C); 1 µM nebivolol induced internalization of the β-ARs with a t½ of 0.6555 min (95% CI: 0.4365 to 1.316 min) and a plateau of 40% of the surface receptors. These data demonstrate that nebivolol induces internalization of β-ARs in a manner that is similar to that of a full agonist. Thus, nebivolol is not a pure antagonist because pure antagonists do not induce receptor internalization. Therefore, either nebivolol is a partial agonist at the β-ARs that leads to robust internalization, a GRK/β-arrestin biased agonist at the β_1_- and β_2_-ARs, or acts as an agonist at an unknown receptor that causes heterologous desensitization of the β-ARs.

**Figure 3:**
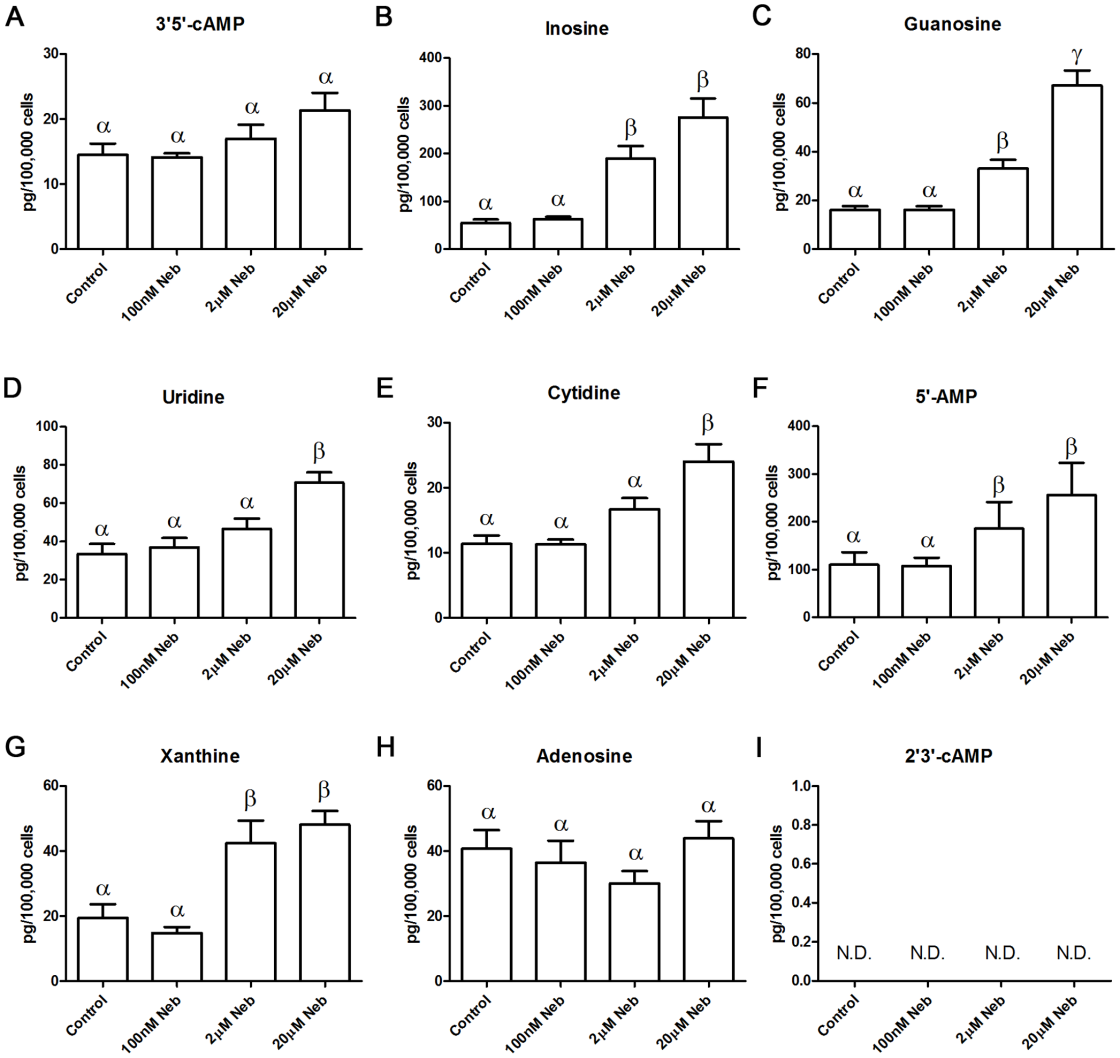
Nebivolol-mediated changes in nucleoside and related compounds concentrations. MEFs were treated with the indicated amount of nebivolol, or vehicle control, for 30 minutes in the presence of IBMX. Each compound was measured from a single sample by LC-MS/MS; the compound examined is listed above each histogram. Bars with different Greek letters are statistically different (p<0.05) from each other according to a one-way ANOVA followed by Tukey-Kramer post hoc test. Data are expressed as mean ± SEM; n = 6.

To begin to determine which of the previous possible explanations describes the observed effects of nebivolol we tested the hypothesis that nebivolol is a partial agonist. If nebivolol is a partial agonist then 3′,5′-cAMP (cAMP from adenylyl cyclase) should increase in a statistically significant concentration-dependent manner. MEFs treated with 100 nM nebivolol for 30 minutes, a concentration that causes internalization, failed to alter 3′,5′-cAMP levels (Fig. 3A); control and 100 nM nebivolol-stimulated levels of 3′,5′-cAMP were (mean ± standard deviation) 14.48±4.39 and 14.12±1.52 (n = 6) pg/100,000 cells, respectively. This result decouples the internalization, which is similar to the full agonist isoproterenol, from cAMP production suggesting that nebivolol is not a partial agonist. On the other hand, 2 and 20 µM nebivolol slightly, and dose-dependently, increased cAMP levels but not to a level that was statistically different from baseline; 2 and 20 µM nebivolol resulted in (mean ± standard deviation) 17.00±5.31 and 21.33±6.65 (n = 6), pg/100,000 cells, respectively. This corresponds to a 1.17 and 1.47 fold increase in 3′,5′-cAMP at 2 and 20 µM nebivolol, respectively. In a separate experiment, MEFs were treated with 10 µM of isoproterenol, which resulted in a greater than 30-fold increase in cAMP levels (data not shown), confirming that we can detect an agonist’s effects on 3′,5′-cAMP, and that the nebivolol-mediated increase in cAMP is exceedingly low. Previous studies indicate that nebivolol has no effect on cAMP [13], [14], including studies using individually expressed β-ARs [9]; the discrepancy between those studies and our data are likely due to two factors: 1) LC-MS-MS detection of 3′,5′-cAMP is the most sensitive technique, and 2) the previous study utilizing cloned β_2_-ARs halted at 1 µM nebivolol and did record a nonsignificant increase in cAMP levels at 1 µM. Collectively the data regarding nebivolol-mediated cAMP production indicates that at concentrations attained in the serum upon clinically used doses of nebivolol, there is no effect on cAMP levels, but at higher concentrations (1 µM and greater) there appears to be marginal cAMP accumulation suggesting that nebivolol may be a weak partial agonist. However, this statistically, and likely biologically, insignificant increase in 3′5′-cAMP does not correlate with, or explain, the internalization data that mirrors a full agonist. This finding suggests that the other mechanisms to explain nebivolol’s effects, biased agonism or a second receptor, are more plausible.

The LC-MS/MS procedure for examining cAMP allows for the quantification of a host of nucleosides and nucleotides, as well as their precursors and metabolites in the same samples [31]. As shown in the rest of Figure 3, nebivolol increased the amount of inosine, guanosine, uridine, cytidine, 5′-AMP, and xanthine in a concentration dependent manner. Nebivolol either had no effect on, or did not statistically increase, the levels of adenosine, 2′-AMP, 3′-AMP, hypoxanthine, thymidine, and uric acid (only adenosine (Fig. 3H) is shown); additionally, 2′,3′-cAMP was not detected (Fig. 3I). 2′,3′-cAMP is generally formed from the breakdown of the poly-A tails on mRNA and is found in abundance in apoptotic cells [39]; therefore, nebivolol did not trigger the degradation of mRNA and is likely not toxic in MEFs. The constellation of nucleosides that were increased suggests that there is increased translation and transcription, which fits with previous studies indicating that nebivolol increases dimethylarginine dimethylaminohydrolase transcript and protein levels [40] and that it alters microRNA expression [41]. Adenosine did not increase, which appears to contradict the translation and transcription hypothesis, yet this may be due to a portion of the increased inosine originating from adenosine degradation. Taken together, the data support previous findings that nebivolol increases translation and transcription. Importantly, all of the statistically different concentration-response changes occurred when there was an absence of a statistically different effect on the cAMP concentration-response. This confirms that nebivolol is not a pure antagonist and demonstrates that nebivolol alters other molecules more potently and efficaciously than 3′,5′-cAMP, indicating that it is not a classical partial agonist, as it does not affect all downstream targets equally, which is the definition of biased agonism.

**Figure 4:**
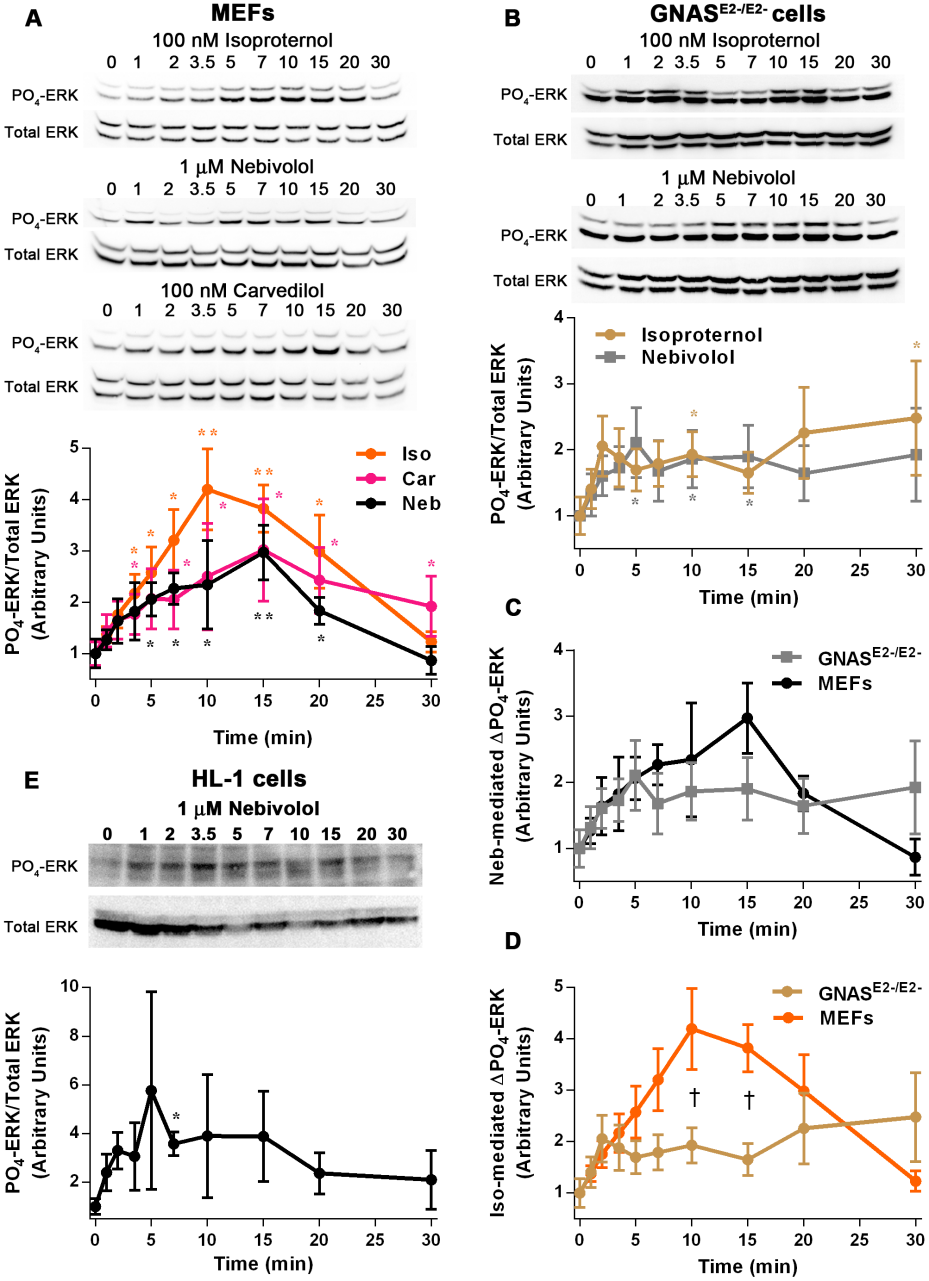
Nebivolol time-dependently and Gα_s_-independently signals to ERK. MEFs were treated with 100 nM of isoproterenol, 1 µM of nebivolol, or 100 nM of carvedilol for the indicated time points (A); representative blots are shown above the graph. Data are compared by one-way ANOVA with Kruskal-Wallis Multiple comparison post-hoc test; two asterisk (**) indicates significantly different than control by Bonferroni method (more stringent) and one asterisk (*) indicates significantly different by the regular test (less stringent). *Gnas* ^E2−/E2−^ cells were treated with 100 nM of isoproterenol and 1 µM of nebivolol for the indicated time points (B); representative blots are shown above the graph. These data were compared as in panel A. The data obtained from the MEFs and *Gnas* ^E2−/E2−^ cells were compared directly for nebivolol- (C) and isoproterenol-mediated (D) phosphorylation of ERK. Data are compared by two-way ANOVA with Sidak’s multiple comparisons post-hoc test; the cross (†) indicates that the MEFs and *Gnas* ^E2−/E2−^ cells are significantly different from each other. HL-1 cardiomyocytes were treated with 10 µM of nebivolol for the indicated time points (E); representative blots are shown above the graph. These data were compared as in panel A. All data are expressed as mean ± SEM; n = 3 to 6.

A common signaling pathway initiated by GRK/β-arrestin biased agonists is the phosphorylation of ERK through transactivation of the EGFR, and EGFR-mediated signaling as well as ERK-mediated signaling is known to increase transcription and translation. To investigate if nebivolol signals in this manner, its time-, receptor-, and GRK-dependent signaling to ERK was examined. First the time course of 1µM of nebivolol-mediated phosphorylation of ERK was compared to 100 nM of isoproterenol and 100 nM of carvedilol (Fig. 4A). Isoproterenol stimulation resulted in a robust phosphorylation of ERK that peaked at 10 minutes and returned to baseline at 30 minutes; whereas nebivolol and carvedilol stimulation increased ERK phosphorylation only half maximally, compared to isoproterenol, and displayed a shallow peak at 15 minutes. The time profile of nebivolol and carvedilol were nearly identical suggesting that they signal through a common mechanism. It should be noted that nebivolol has an approximately 30-fold lower affinity than carvedilol at the β_2_-AR [10], [42], which is why we used greater amounts of nebivolol. Additionally, 10 µM of nebivolol induced a similar time-dependent phosphorylation of ERK as 1 µM of nebivolol (shown with confocal images), except that it appeared to elicit a biphasic response. To further determine if these effects are due to partial agonism at the β_2_-AR, the parental *Gnas* ^E2−/E2−^ cells, which lack all Gα_s_ transcripts [28], were treated with 100 nM of isoproterenol and 1 µM of nebivolol (Fig. 4B). Both isoproterenol and nebivolol stimulation resulted in an approximate 2-fold increase in ERK phosphorylation for the duration of the experiment, which was statistically different from control at multiple points for both agonists. The effects of the two drugs were indistinguishable in the *Gnas* ^E2−/E2−^ cells suggesting that nebivolol and isoproterenol act similarly when the β_2_-AR cannot signal through Gα_s_, and that nebivolol signals to ERK independently of Gα_s_. Nebivolol treatment of MEFs and *Gnas* ^E2−/E2−^ cells yielded results that were not different from each other in a statistically significant manner, but the results did not appear identical (Fig. 4C). The two curves match perfectly for the first 5 minutes, and converge again at 20 minutes. This effect may have been due to differences over time in the cells after stable transfection with Gα_s_ or due to the slight accumulation of cAMP; however, the data clearly demonstrated that Gα_s_ is not absolutely required for nebivolol-mediated signaling. Supporting this conclusion, isoproterenol treatment of MEFs and *Gnas* ^E2−/E2−^ cells were significantly different from each other at 10 and 15 minutes of stimulation (Fig. 4D). To confirm that nebivolol stimulation leads to ERK phosphorylation in a different cell line, HL-1 cells were stimulated with 10 µM of nebivolol (Fig. 4E). HL-1 cells responded more rapidly and more variably than the MEFs with a peak at 5 minutes. These experiments indicate that nebivolol can signal to ERK and that this signaling does not require Gα_s_, thus providing more evidence that nebivolol is not a classical antagonist and that nebivolol fits the profile of a biased agonist. However, these experiments leave open the possibility that nebivolol is acting as an agonist at a novel receptor that causes these effects.

**Figure 5:**
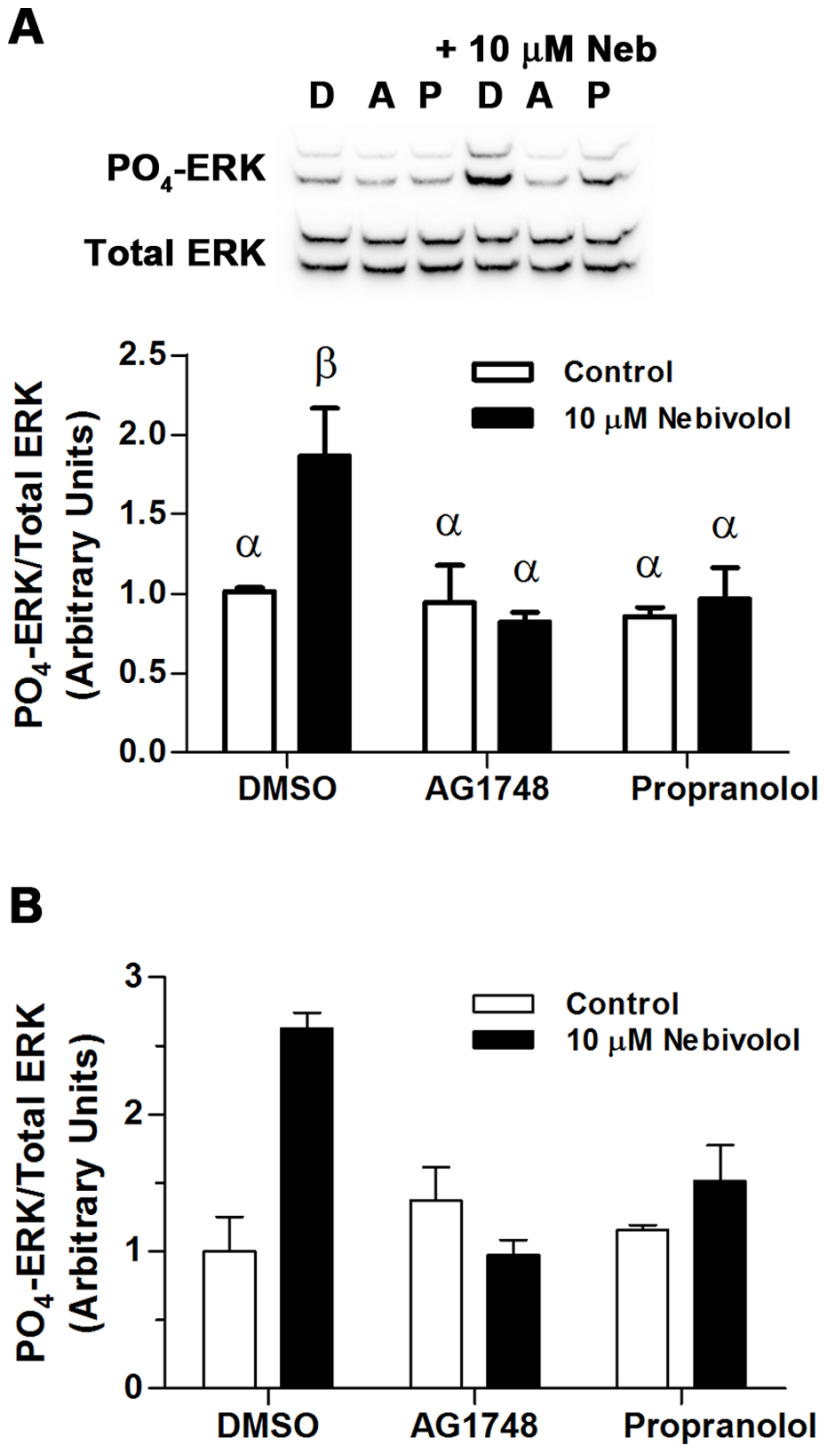
Nebivolol signals to ERK through β-ARs and the EGFR. Cells were pretreated with inhibitors for 30 minutes then stimulated with 10 µM of nebivolol for 7 minutes. Representative blots from MEFs (A) are presented above the histogram of the data; D = DMSO, A = 1 µM of AG1748, and P = 30 µM of propranolol. Open bars are controls, and black bars are nebivolol treated. (B) Identical confirmatory experiments were conducted in two sets of HL-1 cells. All data were analyzed via a two-way ANOVA with Tukey-Kramer post hoc analysis, and significance is denoted by Greek letters. Any data set with a different Greek letter is statistically different p<0.05. Data are expressed as mean ± SEM; n = 4 for MEFs and n = 2 for HL-1 cells.

To examine directly the role of β-ARs, as well as EGFRs, in nebivolol-mediated phosphorylation of ERK, MEFs and HL-1 cells were pretreated for 30 minutes with vehicle, 30 µM of propranolol, or 1 µM of AG1748 prior to stimulation with 10 µM of nebivolol for 7 minutes. Nebivolol-mediated phosphorylation of ERK in MEFs was fully inhibited by both the β-blocker propranolol and the EGFR kinase inhibitor AG1748 (Fig. 5A). This confirms that nebivolol-mediated phosphorylation of ERK emanates from β_2_-ARs and EGFRs in MEFs, similar to what is known for carvedilol [20]. However, this experiment does not distinguish if β_2_-AR activation precedes EGFR activation or vice versa. Yet, it is unlikely that nebivolol is an EGFR agonist. To confirm that these effects are not exclusive to MEFs, a pair of confirmatory experiments was conducted in HL-1 cells (Fig. 5B). The effects in HL-1 cells are indistinguishable from the MEFs, confirming that nebivolol signals through the β-ARs and EGFR and that this signaling is not cell type dependent. Additionally, identical experiments were conducted in MEFs with 1 µM of nebivolol and similar results were obtained (data not shown). These data confirm that nebivolol signals through β_1_- and β_2_-ARs, as well as EGFRs, to ERK. Collectively, these data strongly suggest that nebivolol is a GRK/β-arrestin biased agonist of β_2_- and β_1_-ARs and, like carvedilol, is inducing transactivation of the EGFR; however, these data do not directly examine GRKs or β-arrestin.

**Figure 6:**
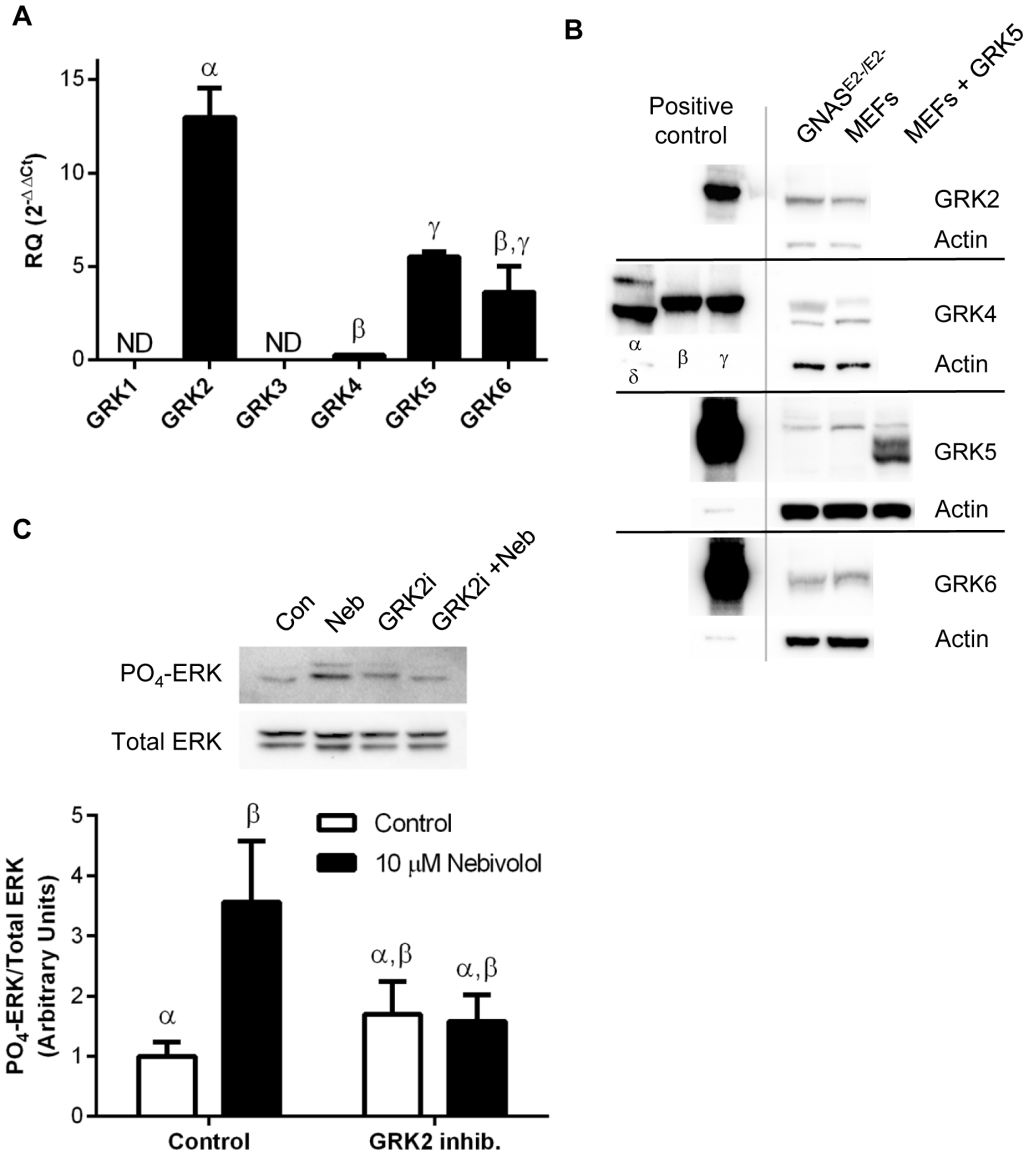
GRK expression and role in nebivolol-mediated phosphorylation of ERK. qPCR was used to identify the GRKs present in MEFs (A); data are expressed as mean ± SEM, n = 3. Western blots for GRK2, 4, 5, and 6 as well as actin, as a control, from GNAS^E2−/E2−^ cells, MEFs, and heterologously expressed GRKs from 293T cells (positive controls) were used to confirm the qPCR data via identifying the protein expression of GRKs (B). MEFs were pretreated with the GRK inhibitor for 30 minutes then stimulated with 10 µM of nebivolol for 7 minutes (C). Representative blots are presented above the histogram of the data. Open bars are controls, and black bars are nebivolol treated. Data are expressed as mean ± SEM; n = 4. All data were analyzed by a one-way ANOVA with Tukey-Kramer post hoc analysis, and significance is denoted by Greek letters. Any data set with a different Greek letter is statistically different p<0.05.

To examine the role of GRKs in nebivolol-mediated phosphorylation of ERK we first identified the isoforms of GRKs expressed in MEFs. qPCR indicates that GRK2 is expressed at levels significantly greater than any other GRK (Fig. 6A). As expected, the negative control, the retinal specific GRK1, is not expressed in MEFs isolated from the torso and appendages of the embryo. Surprisingly, GRK3 message is also not expressed. To confirm that the mRNA levels relate to protein we utilized specific GRK antibodies to detect GRKs 2 through 6 in GNAS^E2−/E2−^ cells and MEFs (Fig. 6B). Cloned GRKs expressed in 293T cells were used as positive controls, and in this process it was found that the GRK3 antibody also detected GRK2 and therefore GRK3 was not included in the analysis. There appears to be much greater protein levels of GRK2 than the GRK4 family; additionally, the bands for GRK5 are higher than expected as shown by stably expressing full length, sequence verified, GRK5 in MEFs. These data support the conclusion from the qPCR data that GRK2 is the most abundant GRK. Fortunately, there is a small molecule inhibitor of GRK2 available [43], allowing for the examination of the role of GRK2 in nebivolol-mediated phosphorylation of ERK. As shown in Figure 6C, 100 µM of the GRK2 inhibitor reduced nebivolol-mediated phosphorylation of ERK. Although nebivolol significantly increases phosphorylated ERK levels, due to the inhibitor alone slightly increasing phosphorylated ERK levels the samples treated with the GRK2 inhibitor were not statistically distinct from nebivolol treatment or the control. However, nebivolol failed to increase phosphorylated ERK levels in the presence of GRK2 inhibitor, supporting the conclusion that nebivolol-mediated phosphorylation of ERK requires GRK kinase activity.

**Figure 7:**
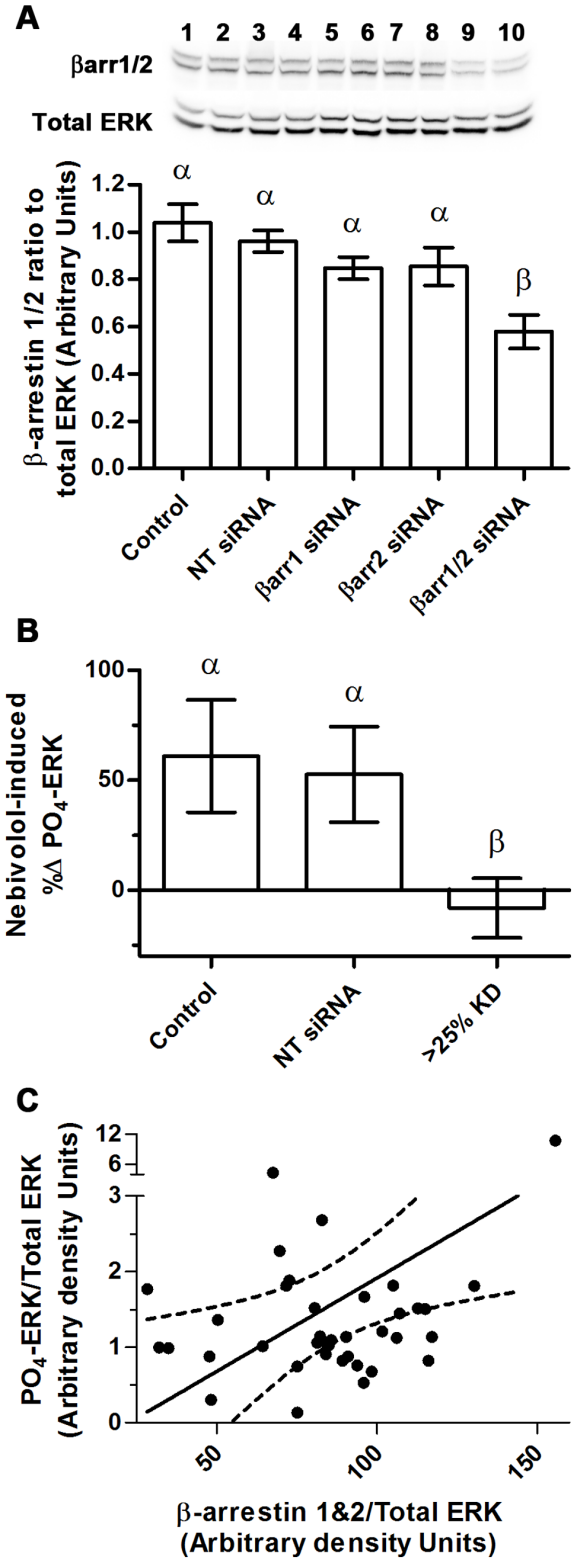
β-arrestins are required for nebivolol-mediated phosphorylation of ERK. siRNA directed towards β-arrestin 1 and 2 were transfected into MEFs; 72 hours later the cells were stimulated with 10 µM of nebivolol for 7 minutes. A representative blot for β-arrestins and total ERK is depicted in panel A above the histogram of the data: even numbers were treated with nebivolol; 1 & 2– non targeting siRNA, 3 & 4– mock transfection, 5 & 6– βarr1 siRNA, 7 & 8– βarr2 siRNA, 9 & 10– βarr1+ βarr2 siRNA. The efficacy of the siRNA was analyzed via a one-way ANOVA with Tukey-Kramer post hoc analysis; statistical difference is denoted by Greek letters; any data set with a different Greek letter is statistically different p<0.05. Data are expressed as mean ± SEM; n = 16. The ERK phosphorylation data (B), collected only from samples where β-arrestins were knocked down by 25% or more, are expressed as the percent change induced by nebivolol. These data were analyzed via a one-way ANOVA with a nonparametric Kruskal-Wallis post hoc test. Statistical difference (p<0.05) is denoted by Greek letters; any data set with a different Greek letter is statistically different. Data are expressed as mean ± SEM; n = 5 to 9. Due to the poor knockdown of β-arrestins, a scatter plot of nebivolol-induced phosphorylation of ERK and β-arrestin level was generated (C) and a Pearson’s correlation was run to determine the relationship between β-arrestin level and nebivolol-mediated phosphorylation of ERK (n = 38); the dotted lines represent the 95% confidence interval of the line.

To determine if there is a role for β-arrestins, siRNA directed against β-arrestins was utilized to knockdown β-arrestin-1 (βarr1) and β-arrestin-2 (βarr2). If nebivolol-mediated phosphorylation of ERK is initiated through β_2_-ARs and involves β-arrestins, then decreases in βarr1 and/or βarr2 should reduce nebivolol-mediated phosphorylation of ERK. To determine if this is the case, siRNA was used to decrease βarr1 and βarr2 separately; however, the siRNA was not specific and minimally reduced the amount of both arrestins (Fig. 7A). Only pooling the βarr1 and βarr2 siRNA resulted in significant knockdown of both β-arrestins. Therefore, the signaling analysis was conducted by pooling the βarr1 and βarr2 data and using a cutoff of 25% knockdown for inclusion in the signaling studies (Fig. 7B). These experiments were conducted identically to the inhibitor studies described in Figure 5 and 6, and the data are expressed as the mean change in nebivolol-induced ERK phosphorylation. In control (no siRNA) and MEFs transfected with non-targeting siRNA, nebivolol stimulation led to increased levels of phosphorylated ERK; however, when β-arrestins were knocked down by 25% or greater, nebivolol, on average, failed to increase phosphorylation of ERK. This finding is consistent with the conclusion that nebivolol is a biased agonist. Since only a fraction of the samples fall within the 25% or more reduction in β-arrestins level, we plotted the level of phosphorylated ERK against the levels of β-arrestin (Fig. 7C). As expected for a GRK/β-arrestin biased agonist, there is a statistically positive Pearson’s correlation (r = 0.3926, P = 0.0148) between the level of β-arrestin and the ability for nebivolol to stimulate ERK.

**Figure 8:**
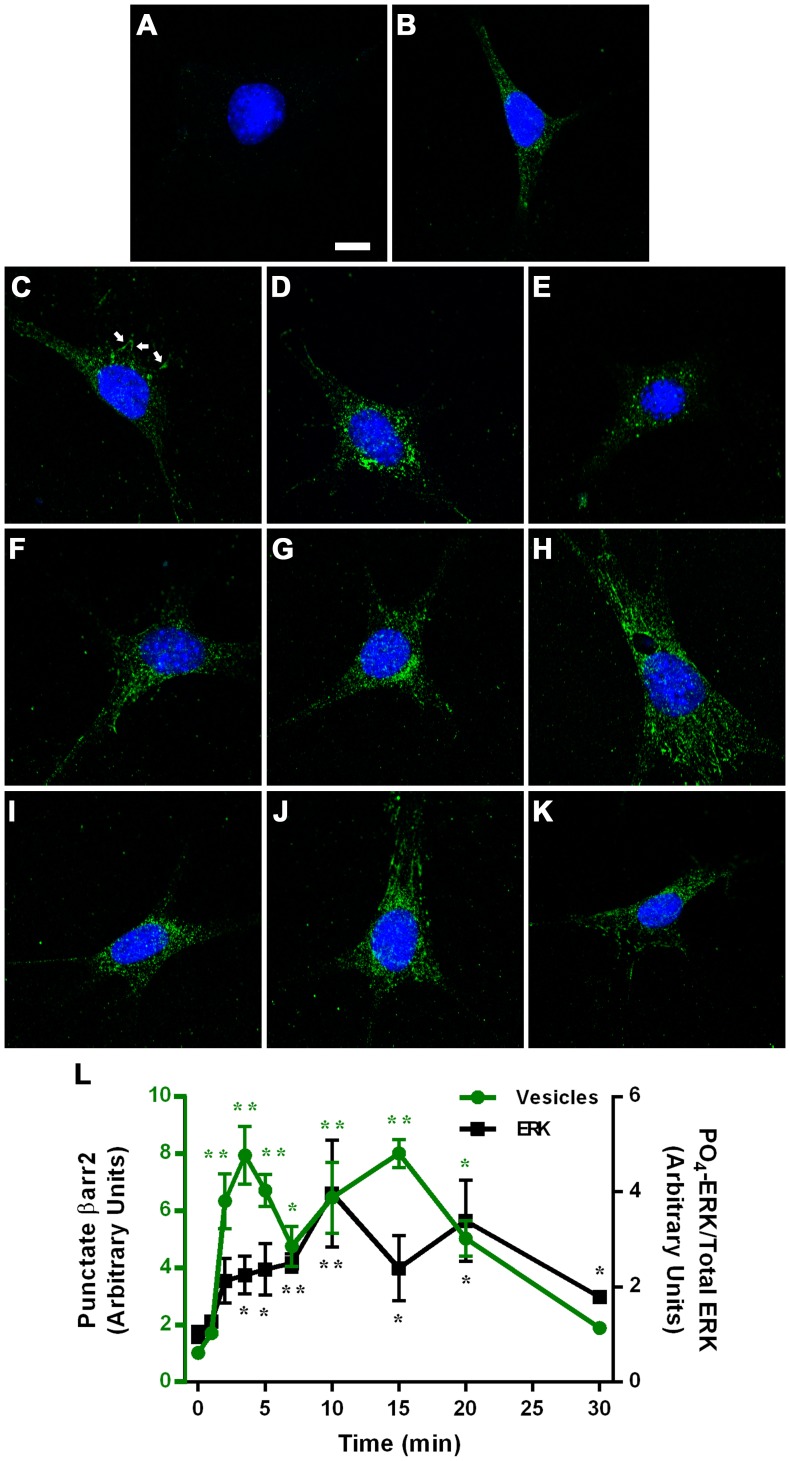
Subcellular distribution of β-arrestin 2 following stimulation with nebivolol. MEFs were treated with vehicle (A and B) or 10 µM of nebivolol for 1 (C), 2, (D), 3.5 (E), 5 (F), 7 (G), 10 (H), 15 (I), 20 (J), or 30 (K) minutes, and stained as described in the methods. Only Alexa 647 secondary antibody and Hoechst 33342 were used in panel A; addition of primary βarr2 antibody (H-9), shown in green, occurred in B through K. All settings remained identical for each image; the scale bar in panel A represents 10 µm. Panel L represents vesicular βarr2 in green on the left axis (n = 8) and 10 µM nebivolol-mediated phosphorylation of ERK in black on the right axis (n = 3 to 7). For both data sets a Kruskal-Wallis Multiple comparison test was run and two asterisk (**) indicates significantly different from control by the Bonferroni method (more stringent) and one asterisk (*) indicates significantly different by the regular test (less stringent).

To confirm that β-arrestins are involved in nebivolol-mediated effects, confocal imaging was used to examine the redistribution of β-arrestins following 10 µM of nebivolol treatment. β-arrestin redistribution occurs when arrestins are activated and can be visualized by the accumulation of β-arrestin at the plasma membrane and/or through accumulation of vesicular structures represented by a punctate staining. Therefore, time dependent β-arrestin redistribution can be used as an assay to identify GRK/β-arrestin biased agonists [44]. Two independent experiments were conducted to examine the cellular distribution of β-arrestins: dual staining for βarr1 and βarr2, and staining for just βarr2 shown in Figure 8. This sequence demonstrated that the antibody used for βarr2 produced a much greater signal than βarr1; the βarr1 signal was too faint to reliably measure over background. As the levels of βarr1 and βarr2 are relatively equal in MEFs as shown in Figure 7A, we attributed the observed difference in staining to the quality of the β-arrestin specific antibodies and therefore only analyzed βarr2 distribution. Very little signal was present with only the secondary antibody (Fig. 8A), whereas diffuse cytoplasmic staining of βarr2 was observed in control cells (Fig. 8B). Treatment with 10 µM of nebivolol for 1 minute had a marginal effect on βarr2 distribution, yet some membrane localization was detectable (Fig. 8C, white arrow represents membrane localized βarr2). Two to 20 minutes of stimulation with nebivolol resulted in a clear redistribution of β-arrestins into punctate structures (Fig. 8D–J); whereas, 30 minutes of stimulation with nebivolol resulted in a staining pattern similar to control and 1 minute stimulation (Fig. 8K). Quantification of vesicle number and intensity shows that nebivolol-mediated redistribution of β-arrestins follows a biphasic curve with a peak at 3.5 and 15 minutes and then approaches the baseline (Fig. 8L). This suggests that the cellular distribution of βarr2 is returning to the basal state 30 minutes after stimulation. Additionally, the biphasic βarr2 redistribution pattern is similar to, but precedes, 10 µM nebivolol-mediated phosphorylation of ERK (Fig. 8L). In accordance with the siRNA depletion of β-arrestins (Fig. 7), the βarr2 peaks preceding the phosphorylation of ERK peaks suggest that nebivolol-mediated βarr2 activation precedes ERK phosphorylation. These data indicate that nebivolol treatment induces redistribution of βarr2 further supporting the conclusion that nebivolol is a biased agonist.

The mechanism(s) via which nebivolol induces signal transduction is of interest due to the ability of nebivolol, a highly specific β_1_-AR antagonist [2], to stimulate NO production [3], [4]. This fundamental contradiction has been baffling and is at least one of the reasons that numerous theories regarding the mechanism of action of nebivolol have been proposed. Herein, we present data that indicates nebivolol is a β_2_-AR, and likely β_1_-AR, GRK/β-arrestin biased agonist. This explains how a β-blocker that is highly specific for the β_1_-AR results in activating signaling pathways without invoking the need for a second receptor. However, it remains an open question if this explains the unique *in vivo* effects attributed to nebivolol [41], [45]. Further studies will have to be conducted to answer this question fully; however, these data present a plausible explanation for nebivolol-mediated signaling, specifically in the endothelium. Most transactivation studies indicate that the ligand activating the EGFR is a heparin-bound EGF-like growth factor (HB-EGF) [46], [47]. Furthermore, HB-EGF stimulates endothelial cell eNOS expression and activity [24] and induces arteriolar vasodilation in a NO-dependent manner [26]. Therefore, it is possible that the biased agonist effect of nebivolol is responsible for generation of NO through transactivation of the EGFR within endothelial cells.

The narrow theory regarding signaling presented above should not be thought of as the only feature of nebivolol. Recent studies utilizing a GRK/β-arrestin biased agonist towards the angiotensin type 1 receptor indicate that GRK/β-arrestin biased agonism leads to phosphorylation of several proteins [48] and that some of the phosphorylation sites are unique to the GRK/β-arrestin biased agonist [49]. Therefore, it is likely that nebivolol shares some signaling pathways with specific β_1_- and/or β_2_-AR agonists, but nebivolol likely induces unique signaling pathways although these pathways still emanate from the β_1_- and/or β_2_-AR. This may have a clinical role; multiple studies indicate that β-arrestins and β-arrestin-mediated signaling are important in cardiac physiology [50], and GRK/β-arrestin biased agonists promote myocyte survival [23], [51]. Therefore, it is likely that the biased agonist property of nebivolol plays a crucial role in its clinical profile.

### Conclusion

Nebivolol is a GRK/β-arrestin biased agonist at the β_2_-AR as demonstrated by the data obtained from the MEFs. It is also likely a GRK/β-arrestin biased agonist at the β_1_-AR as demonstrated by the data obtained from HL-1 cells. These conclusions are supported by the following constellation of results. After 40 cycles of qPCR the β_3_-AR was readily observed in the control but not the MEFs or HL-1 cells. Nebivolol induces β-AR internalization; phosphorylation of ERK through β-ARs, GRK, β-arrestin, as well as the EGFR; redistribution of β-arrestin-2; and it increased nucleoside cellular concentrations without significantly altering cAMP. These effects may explain the recently described physiological differences between treatment with nebivolol and classical β-AR antagonists such as atenolol and metoprolol [41], [45].
